# Thickness Variation of Conductive Polymer Coatings on Si Anodes for the Improved Cycling Stability in Full Pouch Cells

**DOI:** 10.1021/acsami.3c17597

**Published:** 2024-05-15

**Authors:** Philipp Stehle, Frauke Langer, Dragoljub Vrankovic, Montaha Anjass

**Affiliations:** † Institute of Inorganic Chemistry I, Ulm University, Albert-Einstein-Allee 11, D-89081 Ulm, Germany; ‡ Research and Development, Mercedes-Benz Group AG, Mercedesstraße 130/6, 70372 Stuttgart, Germany; § Chemistry of Thin Film Materials (CFTM), IZNF, Friedrich-Alexander University Erlangen-Nürnberg, Cauerstraße 3, 91058 Erlangen, Germany; ∥ Department of Chemistry, University of Sharjah, 27272 Sharjah, United Arab Emirates

**Keywords:** silicon anodes, lithium-ion battery, conducting polymers, solid electrolyte interphase, PEDOT

## Abstract

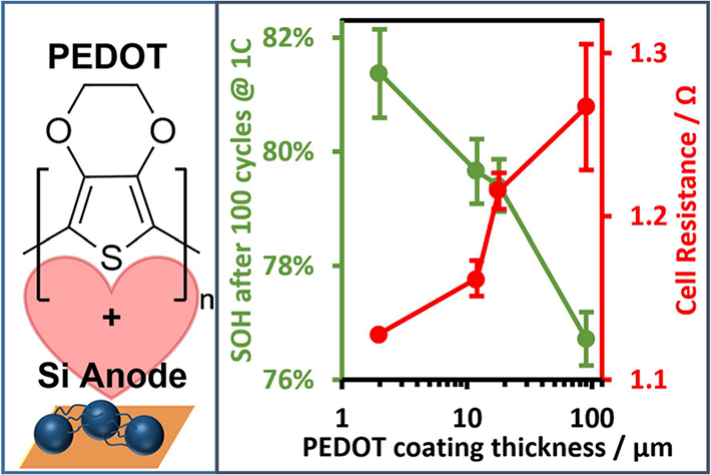

Si-dominant anodes for Li-ion batteries provide very high gravimetric and volumetric capacity but suffer from low cycling stability due to an unstable solid electrolyte interphase (SEI). In this work, we improved the cycling performance of Si/NCM pouch cells by coating the Si anodes with the conductive polymer poly(3,4-ethylenedioxythiophene) (PEDOT) prior to cell assembly via an electropolymerization process. The thicknesses of the PEDOT coatings could be adjusted by a facile process parameter variation. Glow-discharge optical emission spectroscopy was used to determine the coating thicknesses on the electrodes prior to the cell assembly. During electrochemical testing, improvements were observed closely linked to the PEDOT coating thickness. Specifically, thinner PEDOT coatings exhibited a higher capacity retention and lower internal resistance in the corresponding pouch cells. For the thinnest coatings, the cell lifetime was 18% higher compared to that of uncoated Si anodes. Postmortem analyses via X-ray photoelectron spectroscopy and cross-sectional scanning electron microscopy revealed a better-maintained microstructure and a chemically different SEI for the PEDOT-coated anodes.

## Introduction

Lithium-ion batteries (LIBs) have emerged in the past decades as the most suitable technology for portable energy storage applications and electric vehicles.^1−3^ The development of novel cell chemistries is crucial for the improvement of the most important performance indicators, namely, energy density and cycling stability.^4^ On the anode side, commercial LIBs commonly use graphite as the active material, which exhibits high electrical conductivity and cycling stability. However, as the theoretical capacity of graphite is limited to 372 mAh g^–1^, alloying-type anode materials are required to meet the high-energy density targets for the next generation of LIBs.^1,5^ A material of particular interest is Si, which provides a high gravimetric capacity of up to 3579 mAh g^–1^ at room temperature, natural abundance, and low operating voltage (0.2 V vs Li/Li^+^). However, Si suffers from a significantly lower electrical conductivity than graphite (≈10^–3^ vs ≈10^4^ S cm^–1^)^6,7^ and high volume expansion during cycling (more than 300%). The latter causes material pulverization and loss of electrical contact with the current collector.^1,4,8^ Furthermore, the volume expansion leads to continuous fracture of the solid electrolyte interphase (SEI) layer on the active material during cycling. In this context, there is intensive research ongoing regarding the development of Si anodes, on material^9−12^ as well as on electrode and cell levels.^8,13−16^

Conducting polymers (CPs) are intriguing materials for use in Si anodes.^17^ CPs contain a conjugated π–-bond system along their backbone. The overlapping π-molecular orbitals enable electron delocalization along the polymer chains, resulting in an adequate electrical conductivity of these materials.^18,19^ CPs can be applied in various subjects of electrochemistry,^19^ including supercapacitors,^20,21^ solar cells,^22,23^ and fuel cells.^24,25^ In the field of LIBs, CPs have been used in various approaches^18^ together with cathode^26−28^ and anode active materials.^29,30^ The effects on Si anodes have also been widely studied because the CPs can not only act as a conductive additive but also accommodate the volume expansion of Si through their flexible mechanical properties.^17,31,32^ Many studies are focusing on poly(3,4-ethylenedioxythiophene) (PEDOT) and its derivatives because it provides good electrical conductivity of ≈0.1 S cm^–1^ and good electrochemical stability.^33^ In 2012, Cui and co-workers presented a surface coating of PEDOT via a facile electropolymerization process, which significantly enhanced the lifetime of a Si nanowire anode in half coin cells (HCCs).^34^ In this study, we aim to investigate the effects of PEDOT films on Si anodes further under industrially relevant conditions. Regarding surface coatings on Si anodes, prior studies emphasized the critical role of coating thickness in influencing cycle performance.^15,35−38^ The coatings should be uniform and thick enough to enable passivation of the Si surface against detrimental side reactions but not excessively thick to avoid unwanted interfacial resistance and enable fast diffusion of Li ions. Therefore, we aimed to deposit PEDOT films of various thicknesses, expecting differences in their cycling behavior. A nanoporous Si material, which has been introduced and characterized in a previous study,^11^ was used as the active material. We prepared electrodes with 80% Si and an industrially relevant areal capacity of ∼3.0 mAh cm^–2^. The fabricated electrodes were coated with PEDOT layers via a similar process as shown by Cui et al., and the films were characterized via glow-discharge optical emission spectroscopy (GD-OES) and X-ray photoelectron spectroscopy (XPS). Cycle life remains the biggest challenge in the development of Si anodes; therefore, it was selected as the main focus of this study. The pristine and PEDOT-coated Si anodes were tested in an application-oriented full pouch cell setup against NCM811 cathodes. It is important to note that while the electrode and cell design are application-oriented, the study remains at a lab scale. The primary aim of this study was to provide novel insights into the benefits of the different PEDOT film thicknesses on the application-oriented Si anodes, rather than proposing a coating process suitable for industrial applications. During electrochemical testing, thinner PEDOT coatings exhibited higher capacity retention, lower resistance, and higher first cycle efficiency, showing a clear correlation between PEDOT coating thickness and battery performance. For thick PEDOT films, a decrease in capacity was observed compared to uncoated samples, which suggests that an excessively thick coating impedes the access of the active material surface by Li ions. Postmortem analysis via XPS and scanning electron microscopy (SEM) allowed us to gain insight into the anode degradation and SEI formation. These measurements revealed that a chemically different SEI was formed for the PEDOT-coated anodes and their microstructure was better maintained during cycling.

## Experimental Details

### Chemicals and Materials

Nanoporous Si was supplied by E-magy B. V. (the Netherlands). Polyacrylic acid (PAA) had a viscosity average molecular mass (M_v_) of 450,000 g mol^–1^ and was purchased from Sigma-Aldrich (Germany). Graphite was obtained from Showa Denko (Japan), and C45 was obtained from Imerys (France). 3,4-Ethylenedioxythiophene (EDOT) and lithium perchlorate were purchased from Alfa Aesar (US).

### Slurry Preparation and Electrode Casting

The Si anodes were manufactured based on the recipe from Maroni et al.^11^ under an ambient atmosphere using a planetary mixer and a film applicator table. The slurries were prepared to have a content of 80 wt % Si, 10 wt % PAA, 5 wt % graphite, and 5 wt % C45 carbon. For slurry mixing, a 10 wt % solution of PAA was prepared and a blend of the powders, as well as water, was added subsequently with mixing steps in between to maintain a liquid phase of medium viscosity. The slurry was cast onto stainless steel foil (litarion, 10 μm) using a BYK doctor blade and a casting speed of 1 cm s^–1^. Stainless steel was used as the current collector because a partial dissolution of copper foil was observed in the PEDOT electropolymerization solution. The casted electrodes were dried at 65 °C under ambient pressure until visibly dry and afterward for another 45 min at 65 °C under vacuum. Target loadings of the electrodes were between 1.48 and 1.52 mg_Si_ cm^–2^.

### Electropolymerization of PEDOT on Si Anodes

The electropolymerization was based on the process used by Cui et al.^34^ A two-electrode configuration was used with Pt as the counter electrode and a punch of the prepared Si electrode (11.34 cm^2^ coated area) as the working electrode. EDOT (0.01 M) and lithium perchlorate as a supporting electrolyte salt (0.1 M) were dissolved in acetonitrile. While stirring the solution with a magnetic stirrer, chronopotentiometry was used to constantly apply the current over the corresponding amount of time (see Table 1). After this step, the Si electrodes were washed thoroughly with acetonitrile to remove residual electrolyte salt and monomer and then dried in an ambient atmosphere to remove the residual solvent. For testing in full pouch cells, the PEDOT-coated electrodes were used directly in the same format.

**Table 1:** Applied Parameters during the Electropolymerization Process for PEDOT Coatings with Different Thicknesses on the Si Anodes^a^

label	approx. PEDOT film thickness (nm)	applied current (mA/cm^2^)	reaction time (min)
thick	80–100	1.0	2
medium	18–22	0.3	2
thin	12–15	0.1	2
very thin	2–3	0.1	0.5

a Different thickness descriptions used in the course of this paper refer to these conditions. The thickness values stem from the GD-OES measurements shown in Figure 2b. Please note that an exact determination of the PEDOT coating thickness is challenging due to the porosity of the electrode.

### Full Pouch Cell Assembly and Cycle Life Tests

Double-sided cathodes with a loading of ∼3.0 mAh cm^–2^ per side and a formulation of 94.5% commercial NMC811, 1 wt % C65 carbon, 1 wt % multiwalled carbon nanotubes, and 3.5 wt % polyvinylidene fluoride were used as the positive electrodes. One cathode per cell was placed in between two Si anodes, separated by ceramic-coated polyethylene separator of 21 μm thickness. The capacity of the Si anodes was limited to 2000 mAh g^–1^, steered by the negative:positive electrode ratio of the cells. The coated electrode area was 11.34 cm^2^ for the Si anodes and 10 cm^2^ for the NMC811 cathodes. After drying the electrode stacks at 80 °C under vacuum overnight and filling with 0.5 mL of electrolyte, the pouch cells were pressed between acrylic glass plates using 3-fold-back clamps and allowed to rest for 2 h. For formation, the cells were subsequently galvanostatically cycled at 25 °C between 2.8 and 4.2 V for two C/10, two C/5, and three C/3 cycles, subsequently. For the long-term cycling evaluation, 1C cycles were applied after the formation protocol until a state of health (SOH) of 80% was reached. After each charge step at constant current (CC), a constant voltage phase was applied with a cutoff current equal to 10% of the preceding CC. At the beginning of the cycling program and after every 50 cycles, a C/3 checkup cycle was applied. These checkup cycles included evaluation of the current state of health (SOH) of the cell and a pulse test to determine the direct current internal resistance (DCIR). The pulse was applied for 5 s with a current rate of 1C in the discharging direction.

### Postmortem Electrode Treatment

Postmortem electrode samples for analytics were prepared by first recovering the anode from the pouch cell under an Ar atmosphere, washing it for 30 s in a bath of dimethyl carbonate, and allowing the solvent to evaporate at ambient temperature in an Ar atmosphere. Inert sample transfer between gloveboxes was carried out by sealing the sample-containing vials in small pouch bags under an Ar atmosphere.

### Scanning Electron Microscopy (SEM)

SEM samples were prepared by cross-sectional milling of the anodes using a Hitachi IM 4000 Plus ion polisher. Images were taken on a Tescan MIRA 3 microscope using a voltage of 15 kV electron beam.

### Glow-Discharge Optical Emission Spectroscopy (GD-OES)

GD-OES measurements were performed using a GDA750 spectrometer (Spectruma) with Argon 5.0 as discharge gas. An air-sealed transfer chamber was used for all of the measurements. The analyzed spot size is 2.4 mm in diameter. For GD-OES calibration, nine electrode samples were produced with silicon contents of 0.66–73.53 wt % of silicon in a graphite matrix. A silicon sputtering target was used as 100% silicon sample. To take matrix effects into account and enable quantification of sulfur in a silicon matrix with high precision, five electrode samples with lithium bis(trifluormethylsulfonyl)amid as an additive in a silicon matrix were produced, resulting in sulfur contents of 0.30–2.52 wt %. Calibration samples were characterized by photometric silicon quantification and elemental analysis.

### X-ray Photoelectron Spectroscopy (XPS)

A commercial XPS machine from Physical Electronics (PHI 5800 ESCA) equipped with a hemispherical electron analyzer, a monochromatic Al K_α_ X-ray source (1486.6 eV), and a flood gun to avoid charging of the sample was used for the measurements. Survey and detail spectra were recorded using pass energies at the analyzer of 93.9 and 29.35 eV, respectively. Both angles (angle of photon incidence on the sample and angle of emitted photoelectrons) are 45° with respect to the surface normal (sample holder, respectively). The binding energies (BEs) of all spectra were calibrated with respect to the C 1s peak of ubiquitous carbon, which was fixed at a binding energy (BE) of 284.7 eV. The data were evaluated (deconvolution of spectra) by using the commercial software package CasaXPS (Casa Software Ltd., version 2.3.23PR1.0). In the first step, Shirley background subtraction was performed.

## Results and Discussion

**Figure 1:**
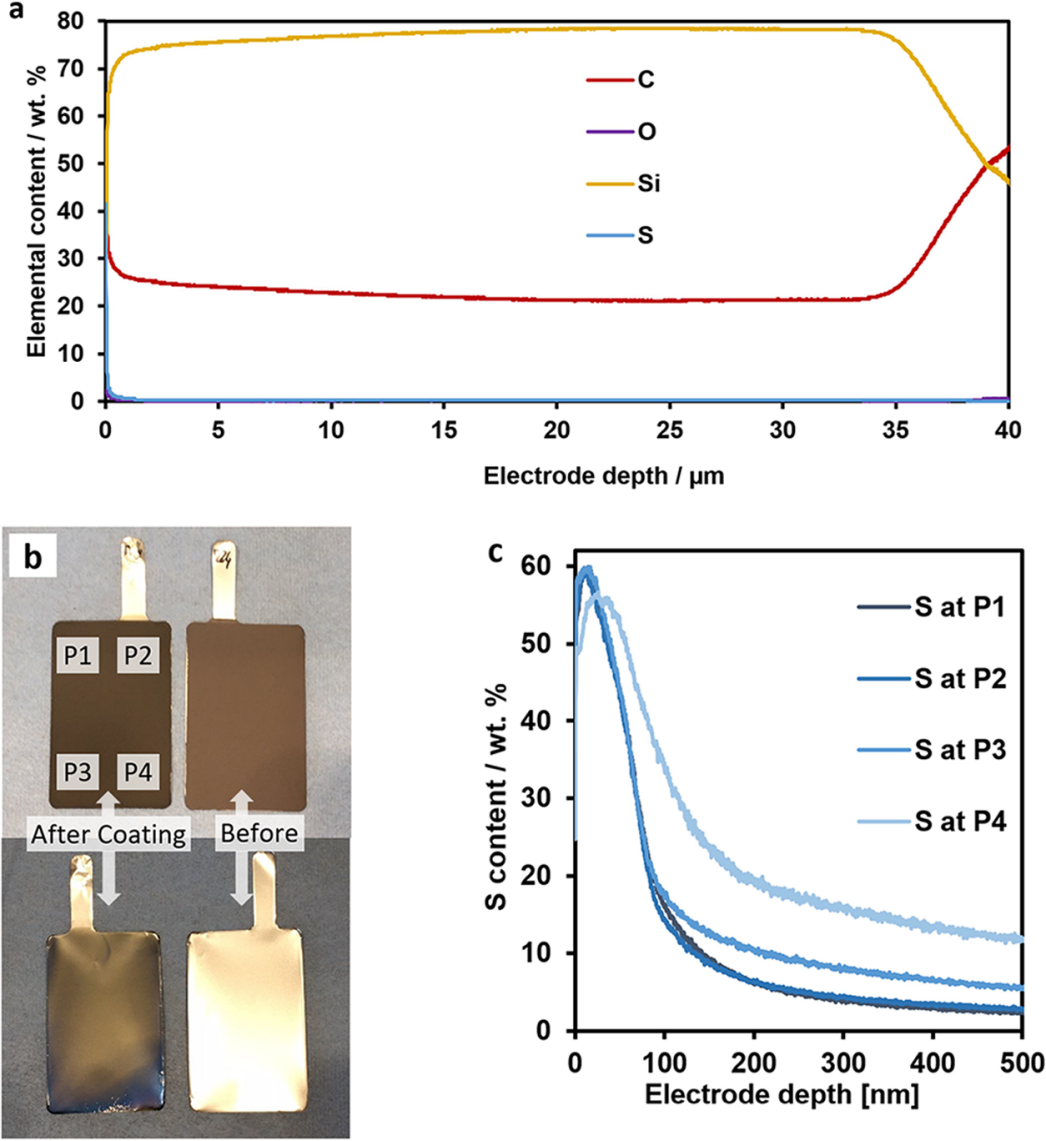
Exemplary complete glow-discharge optical emission spectrum of a PEDOT-coated Si anode (a), photos of a PEDOT-coated compared to a pristine anode from front and back (b), and excerpt from glow-discharge optical emission spectra measured at the four different positions of a PEDOT-coated anode (c). The data in this picture originate from anodes coated with 1 mA/cm^2^ for 2 min. Coatings obtained from these conditions are referred to as “thick” in the following figures.

For the first coating, we used identical conditions as described by Cui et al. and applied 1 mA/cm^2^ for 2 min between the working and counter electrodes. The as-deposited PEDOT films were characterized via GD-OES and XPS measurements. Figure 1 shows the obtained data for the PEDOT-coated Si anodes.

As shown in Figure 1b, a color change on both sides of the Si anode was observed, which is assigned to the formation of the characteristic blue PEDOT layer, thus being the first indication of a successful coating. Figure 1a shows the corresponding GD-OES sputtered through the complete depth of the anode. It exhibits increased S and C concentration during the first ∼100 nm, which is assigned to the PEDOT polymer film. For the following evaluations, the S concentration is used as a marker for PEDOT, as it is the only exclusive heteroatom in the polymer. After a depth of ∼1 μm in the anode, the Si concentration is rising to an almost constant value of ∼78 wt %, while the concentration of C drops to ∼21 wt %. These values are in accordance with expectations based on the formulations of the electrode. After ∼35 μm in the electrode, the Si concentration decreases and the C concentration increases significantly. This indicates that the bottom end of the anode coating (consisting of Si active material, binder, and conductive additive) is reached and the stainless steel current collector is measured. For the further GD-OES measurements, we narrowed the analytical depth down to 1.5 μm of electrode depth because we demonstrated that the PEDOT film thickness could be well determined in that way. Four different spots of a single PEDOT-coated Si anode were measured to verify the homogeneity of the coating. The obtained S spectra (depicted in Figure 1c) show that thickness variations occur in the coating, particularly on the bottom right spot. We assign this observation to the asymmetry of the electrode, resulting in higher local current densities at position P4. The thickness of the surface film was determined to be in the range 80–100 nm for electropolymerization with 1 mA/cm^2^. Figure S1 shows X-ray photoelectron spectra measured on the surfaces of the PEDOT-coated compared to the pristine Si anode. The latter exhibits C (1s) and Si (2p) peaks, which originate from the electrode components (Si active material, graphite, carbon black, and binder) and an O (1s) peak, which we assign to a silicon oxide film naturally formed on the active material surface. By comparison, the spectrum of the PEDOT-coated anode shows additional S (2p) and Cl (2p) peaks, which can be assigned to the PEDOT film and its ClO_4_^–^ dopant. Accordingly, the intensity of the C (1s) peak is slightly higher than that in the pristine anode spectrum. The Si (2p) peak is negligibly small in the spectrum of the PEDOT-coated sample, confirming complete coverage of the Si particles by the PEDOT film. Combined, the GD-OES and XPS results prove that the PEDOT coating on the Si anode had acceptable homogeneity. The subtle thickness variation observed across the PEDOT coating highlights the importance of ongoing efforts to improve the coating process for better uniformity.

**Figure 2:**
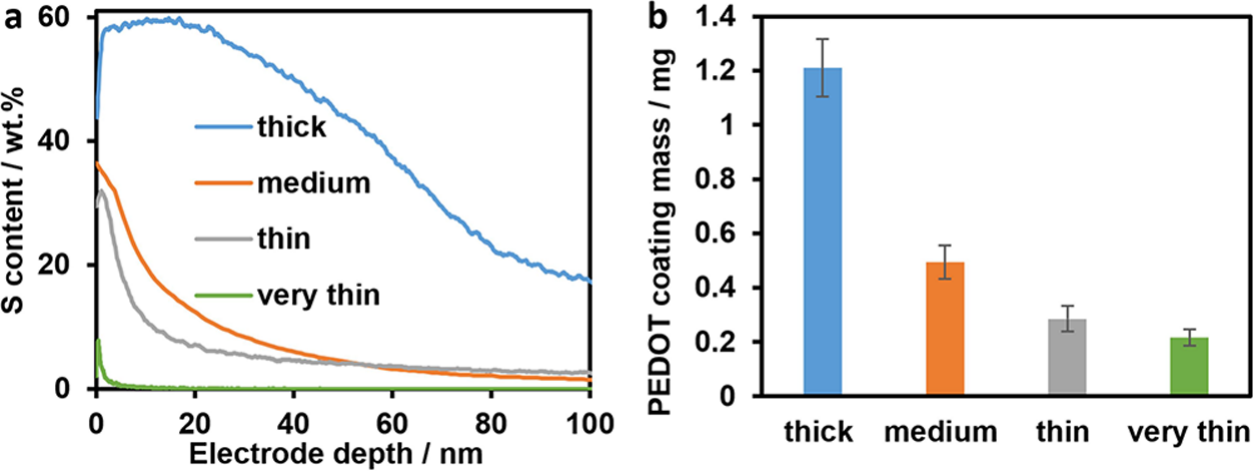
Glow-discharge optical emission spectra of Si anodes coated with different thicknesses of PEDOT (a) and net masses of the corresponding PEDOT coatings (b).

Figure 2a shows the GD-OES spectra comparing the coatings we obtained using different current densities and reaction times during the electropolymerization process (see Table 1 for the exact conditions). As expected, thinner PEDOT coatings were obtained when applying lower current densities and shorter reaction times. The thick coating corresponds to the measurement shown in Figure 1c, being ∼ 80 to 100 nm in thickness. For the coatings labeled as medium and thin in Table 1, films in the ranges of ∼18 to 22 and ∼12 to 15 nm were obtained, respectively. When applying the conditions for the very thin coating, the resulting film was just ∼2 to 3 nm thick. In line with expectations, the mass of the PEDOT coatings (determined by weighing the electrodes before and after the coating process) also decreased when lower current densities and reaction times were applied, as shown in Figure 2b. These findings prove that the applied current density and reaction time during electropolymerization highly influence the thickness of the PEDOT films and we could obtain coated Si anodes of four different thicknesses.

As shown in Figure S2a, a decreased capacity is observed for the thickest PEDOT coating during the cell formation cycles compared to the pristine and all other PEDOT-coated anodes. With this film being significantly thicker and broader distributed compared to the others, these results indicate that pores of the Si anode are blocked by the PEDOT coating, reducing the Li-ion pathways. This assumption is supported by the significantly lower first cycle Coulombic efficiency observed for the thickest coating (77.1 ± 0.4 vs 80.0 ± 0.1% for the pristine anode, shown in Figure S2b). For the other PEDOT coatings, the average Coulombic efficiency in the first cycle increases for lower thicknesses. This is assigned to an “artificial SEI” effect provided by the PEDOT layer, which partially suppresses side reactions on the anode surface during the first cycle.

**Figure 3:**
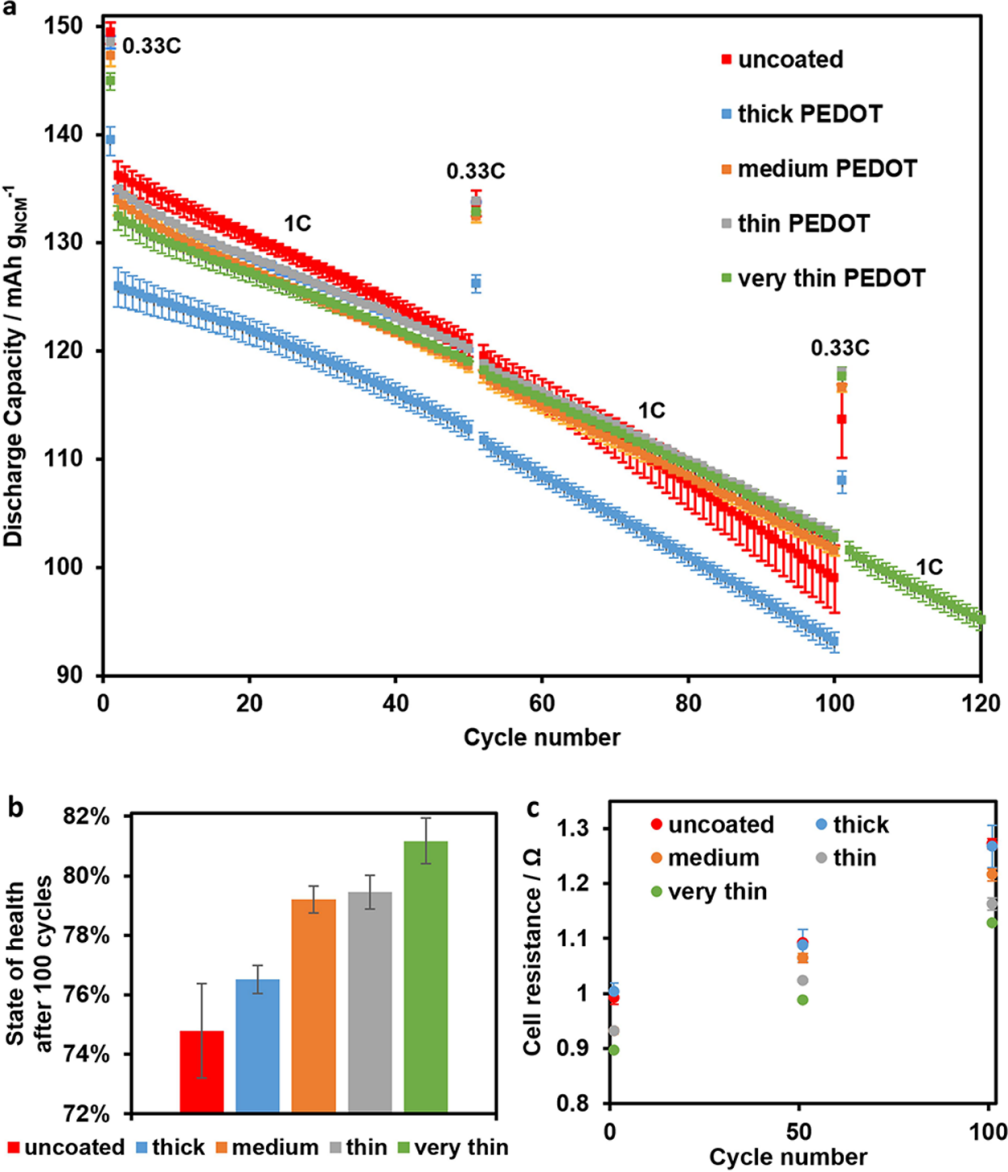
Discharge capacities obtained during the cycling protocol (a), corresponding capacity retentions after 100 cycles related to 1C (b), and internal resistances measured 5 s after the discharge pulses (c).

Figure 3 shows the data obtained during the cycling protocol. For all PEDOT-coated Si anodes, an improvement in capacity retention was observed compared with the pristine Si anodes. The effect becomes more significant for thinner coatings. As shown in Figure 3a, the uncoated Si anodes exhibit the highest discharge capacity in the first C/3 cycle. However, after 100 cycles at 1C, a higher capacity is observed at C/3 for all PEDOT films except the thickest (coated at 1 mA/cm^2^). Figure 3b shows the obtained state of health (SOH) values after these 100 cycles related to the first 1C cycle. A significant increase of ∼7% is found for the very thin coating (78.0 vs 72.8% for the pristine Si anode). The corresponding cells underwent 13 cycles more until the SOH of 80% was reached at 1C, which equals an increase of ∼18% in cycle life. As shown in Figure 3c, we measured a significantly lower DCIR for some of the thinnest PEDOT coatings compared to those of the pristine anodes, especially in the later cycles. These findings suggest that the PEDOT layer helps to maintain the microstructure of the anode, mitigating the loss of electrical contact between the particles. The influence of PEDOT film thickness is crucial, which is highlighted by the poor performance of the thickest coating. It is hypothesized that an excessively thick PEDOT coating partly blocks the Si surface and impedes Li-ion diffusion, causing reduced capacity and higher resistances in the anode. Accordingly, the cells with the thinnest PEDOT coatings exhibit the highest capacity retention and lowest internal resistance. Overall, the improvement in cycle life is less significant compared to the findings of Cui et al., which is assigned to the completely different microstructure of the anode. In their nanowire system, the pores between Si are much larger compared to our anode consisting of microsized particles. This potentially allows the PEDOT films to distribute deeper into the electrode and cover more active material surfaces, therefore exerting its advantages more significantly.

**Figure 4:**
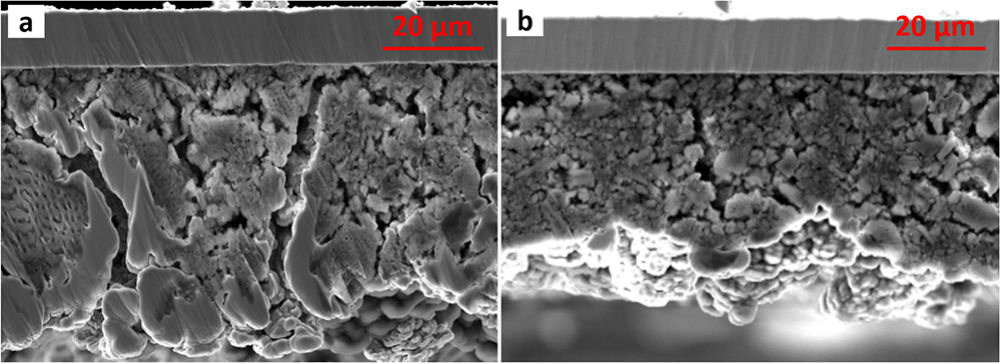
Cross-sectional SEM images of postmortem Si anodes after 100 cycles without PEDOT coating (a) and with a PEDOT coating obtained after applying 0.1 mA/cm^2^ for 0.5 min (b).

We conducted measurements via SEM and XPS on the thinnest (and most effective) PEDOT-coated Si anodes after 100 cycles and compared them to those of pristine Si anodes, which underwent the same number of cycles. The cross-sectional SEM images shown in Figure 4 prove that the microstructure of the PEDOT-coated Si anode is much better maintained compared to the uncoated Si anode after 100 cycles. Many microsized cracks, reaching through the complete anode layer, can be seen in the image of the uncoated anodes. In the PEDOT-coated anode, fewer cracks are observed, which are much narrower and less deep. This trend was reported by Cui et al. as well and supports the assumption that PEDOT helps to increase the particle adhesion mechanically and electronically through its flexible, conductive character.

**Figure 5:**
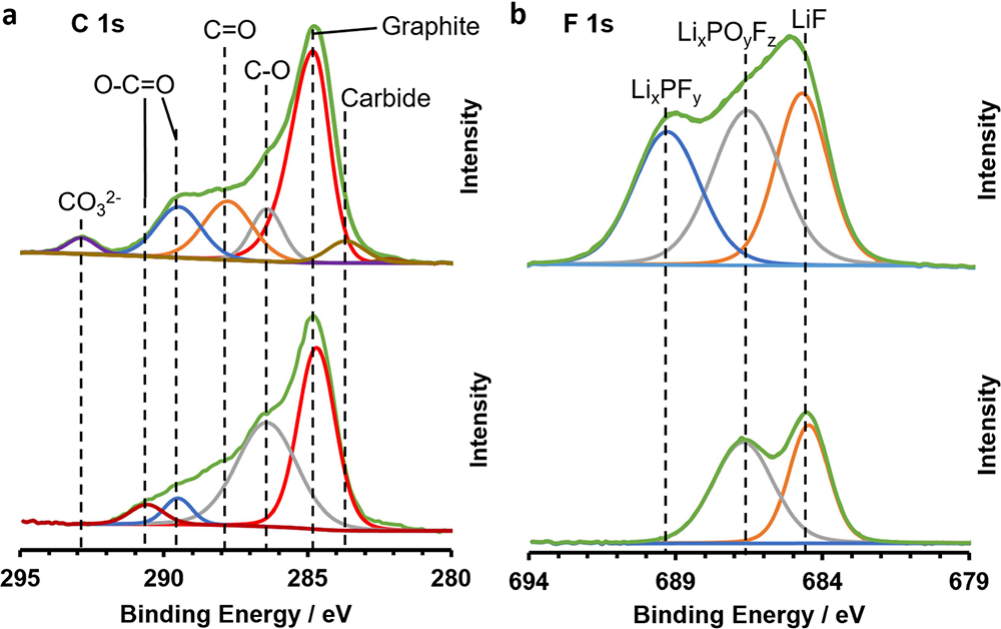
F1 s (a) and C1 s (b) X-ray photoelectron spectra of postmortem Si anodes after 100 cycles without PEDOT coating (top) and with a PEDOT coating (bottom) obtained after applying 0.1 mA/cm^2^ for 0.5 min.

XPS measurements were carried out on the cycled anode surfaces to gain insight into the SEI. The corresponding spectra are shown in Figure 5. In the C 1s spectra in Figure 5a, reduced amounts of C=O, O–C=O, and CO_3_^2–^ species are observed in the PEDOT-coated compared to the uncoated anode. These species can be assigned to lithium alkyl carbonates, which are known to be decomposition products of the electrolyte solvent ethylene carbonate (EC).^39,40^ Meanwhile, C–O species exhibit an increased amount in the PEDOT-coated anode. These groups are characteristic for the polymerization products of the additive vinylene carbonate (VC), which have been reported to improve the SEI of Si anodes.^41^ In the F 1s spectra in Figure 5b, the amount of Li_*x*_PF_*y*_ and Li_*x*_PO_*y*_F_*z*_ is significantly decreased in the PEDOT-coated anode compared to that in the uncoated anode. Such P–F species are known as the decomposition and hydrolysis products of the conductive salt LiPF_6_. The signal originating from LiF, a typical reaction product of the additive fluoroethylene carbonate (FEC),^40,42^ is slightly lower in the PEDOT-coated anode as well. LiF is known as beneficial for the SEI stability of Si anodes; however, the other SEI modifications caused by PEDOT seem to outweigh this effect. In total, the XPS results demonstrate that the PEDOT film influenced the chemical composition of the SEI. Unfavorable decomposition reactions of LiPF_6_ and EC were suppressed. Meanwhile, the level of polymerization of the beneficial additive VC seems to be proportionally higher. It can be hypothesized that the resulting SEI composition contributed to the improvement in the cycling stability observed in the corresponding cells.

## Conclusions

In summary, we applied conductive PEDOT coatings on porous Si-dominant anodes via a facile electropolymerization process. Variation of the applied current density and reaction time resulted in four different thicknesses of the PEDOT layers. Slight thickness variations could be observed across the coatings. While a significantly decreased capacity was observed for the anodes with the thickest PEDOT layers in Si/NCM pouch cells, the thinner films exhibited a similar capacity compared to that of the uncoated anodes. With a decrease in the PEDOT layer thickness, the first cycle Coulombic efficiency and cycling stability of the respective cells increased, while the internal cell resistance was reduced. For the thinnest layer (∼2 nm according to GD-OES measurements), improvements in capacity retention of ∼7% and cycle life of ∼18% compared with the uncoated Si anodes were obtained. The results demonstrate that CP films can help improve the cycling stability of porous Si anodes in full cells, with the thickness of the coating having a big influence on the cycling performance. Consecutive studies should focus on further improving the process for a better PEDOT coating uniformity.

## Supporting Information Available

Complete XPS spectra of pristine and PEDOT-coated Si anodes, discharge capacity comparison during cell formation, first cycle efficiencies, and SEM-EDS mapping of the PEDOT-coated Si anode surface (PDF)
