# Supplementary material for: Thickness Variation of Conductive Polymer Coatings on Si Anodes for the Improved Cycling Stability in Full Pouch Cells
Source: ACS Appl Mater Interfaces. 2024 May 15;16(21):27202–8. doi: 10.1021/acsami.3c17597 (PMC11145580; doi:10.1021/acsami.3c17597)
Supplement: Supplementary file 1 — am3c17597_si_001.pdf [file am3c17597_si_001.pdf]

Supporting Information

Thickness variation of conductive polymer coatings on Si anodes for the improved cycling stability in full pouch cells

Philipp Stehle[a,b], Frauke Langer[b,c], Dragoljub Vrankovic[b], Montaha Anjass*[a,d]*

[a] Institute of Inorganic Chemistry I,

Ulm University, Albert-Einstein-Allee 11, D-89081 Ulm, Germany

[b] P. Stehle, F. Langer, D. Vrankovic; Research and Development,

Mercedes-Benz Group AG, Mercedesstraße 130/6, 70372 Stuttgart, Germany

[c] F. Langer; Chemistry of Thin Film Materials (CFTM), IZNF,

Friedrich-Alexander University Erlangen-Nürnberg; Cauerstraße 3, 91058 Erlangen, Germany

[d] M. Anjass; Department of Chemistry,

University of Sharjah, Sharjah-27272, Sharjah, United Arab Emirates

*Corresponding authors: P. Stehle, M. Anjass

E-mails: philipp.p.stehle@daimlertruck.com, montaha.anjass@uni-ulm.de

Supporting Figures

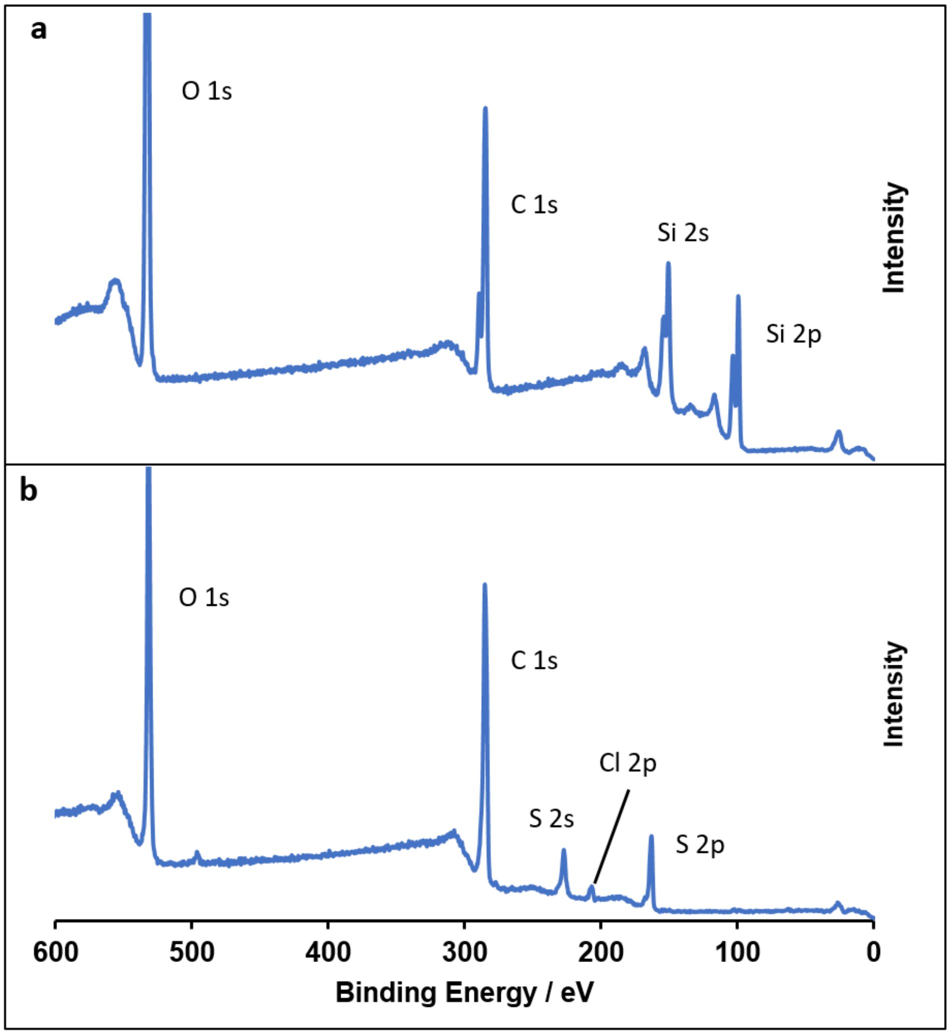

Figure S1. X-ray photoelectron spectra of a pristine (a) and PEDOT-coated Si anode (b). The S and Cl signals originate from the PEDOT film.

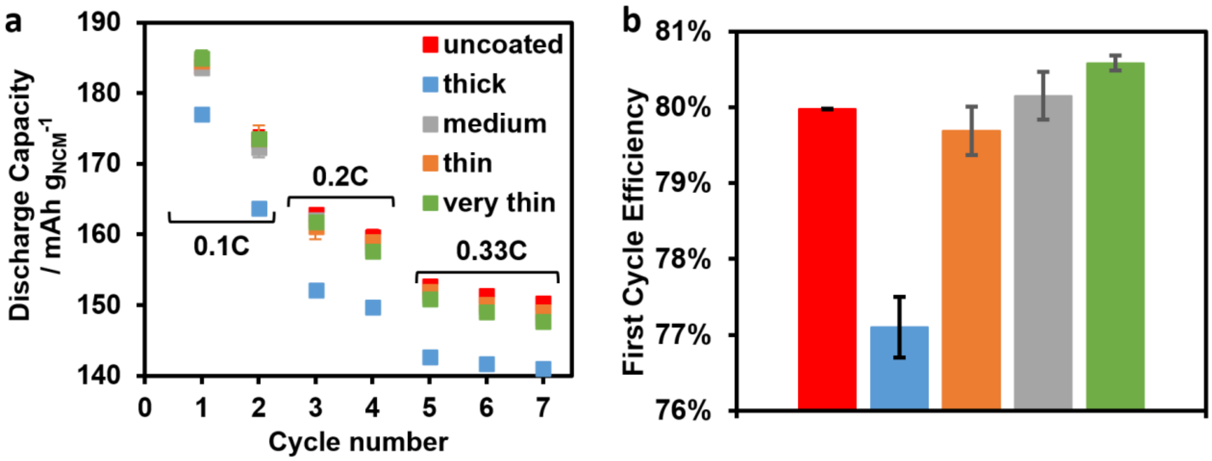

Figure S2. Discharge capacities (a) and coulombic efficiencies of the first cycle (b) for uncoated and PEDOT-coated Si anodes, obtained during the formation cycles.

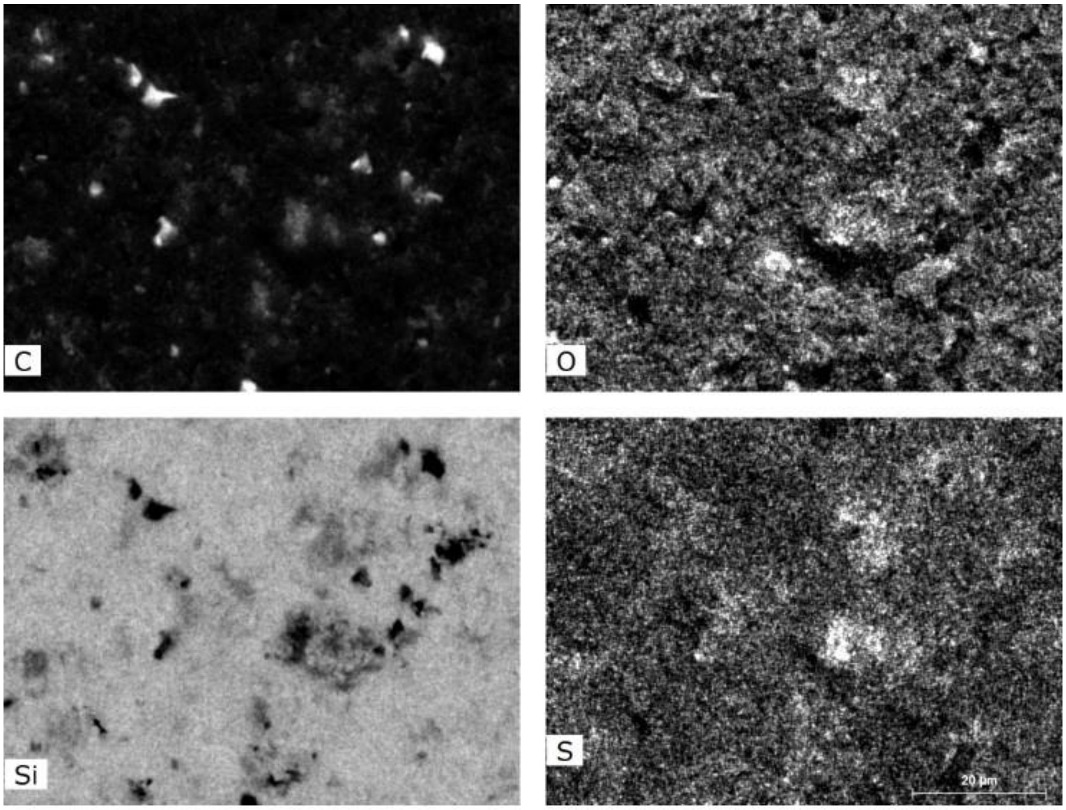

Figure S3. SEM-EDS mapping of the surface of a PEDOT-coated Si anode.

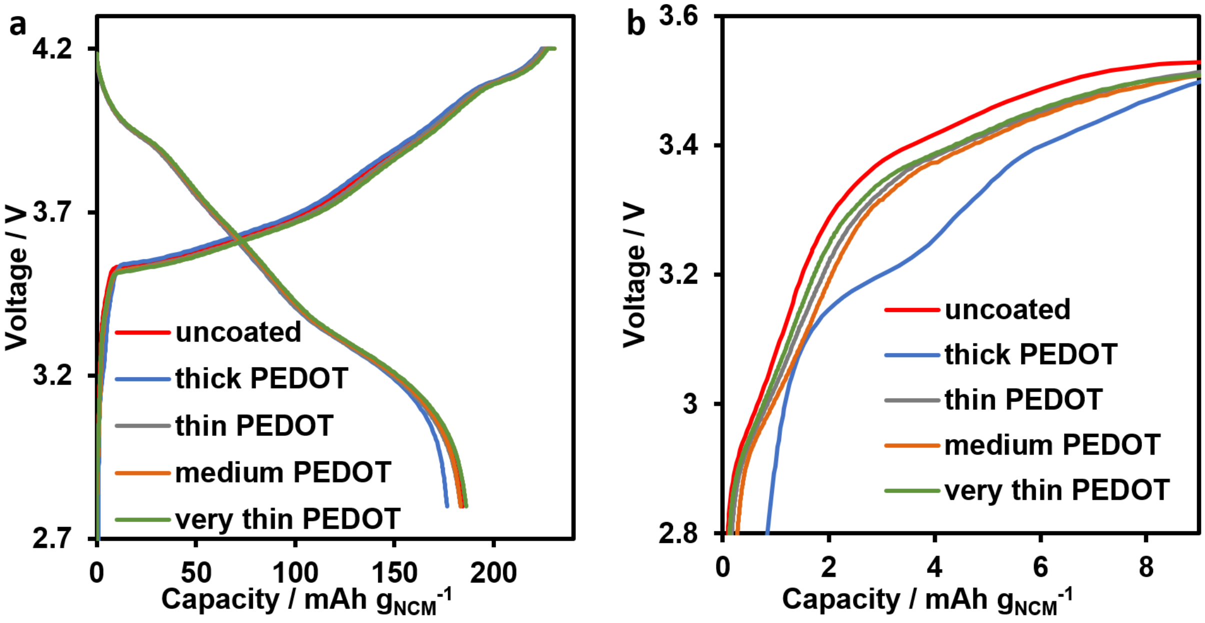

Figure S4. Voltage curves of charge and discharge obtained for uncoated and PEDOT-coated Si anodes during the first formation cycle (a) and enlarged excerpt from the charge curves (b). An influence of the PEDOT coating thickness on the charge voltage curve and thus the SEI formation is visible.
